# How Do Plants Respond to Combined Drought and Salinity Stress?—A Systematic Review

**DOI:** 10.3390/plants11212884

**Published:** 2022-10-28

**Authors:** Prodipto Bishnu Angon, Md. Tahjib-Ul-Arif, Samia Islam Samin, Ummya Habiba, M. Afzal Hossain, Marian Brestic

**Affiliations:** 1 Faculty of Agriculture, Bangladesh Agricultural University, Mymensingh 2202, Bangladesh; 2 Department of Biochemistry and Molecular Biology, Bangladesh Agricultural University, Mymensingh 2202, Bangladesh; 3 Institut of Plant and Environmental Sciences, Slovak University of Agriculture, A. Hlinku 2, 94976 Nitra, Slovakia

**Keywords:** abiotic stress, antioxidants, combined stress, ionic homeostasis, photosynthesis, plant growth, osmotic stress, salt stress

## Abstract

Plants are frequently exposed to one or more abiotic stresses, including combined salinity-drought, which significantly lowers plant growth. Many studies have been conducted to evaluate the responses of plants to combined salinity and drought stress. However, a meta-analysis-based systematic review has not been conducted yet. Therefore, this study analyzed how plants respond differently to combined salinity-drought stress compared to either stress alone. We initially retrieved 536 publications from databases and selected 30 research articles following a rigorous screening. Data on plant growth-related, physiological, and biochemical parameters were collected from these selected articles and analyzed. Overall, the combined salinity-drought stress has a greater negative impact on plant growth, photosynthesis, ionic balance, and oxidative balance than either stress alone. In some cases, salinity had a greater impact than drought stress and vice versa. Drought stress inhibited photosynthesis more than salinity, whereas salinity caused ionic imbalance more than drought stress. Single salinity and drought reduced shoot biomass equally, but salinity reduced root biomass more than drought. Plants experienced more oxidative stress under combined stress conditions because antioxidant levels did not increase in response to combined salinity-drought stress compared to individual salinity or drought stress. This study provided a comparative understanding of plants’ responses to individual and combined salinity and drought stress, and identified several research gaps. More comprehensive genetic and physiological studies are needed to understand the intricate interplay between salinity and drought in plants.

## 1. Introduction

Drought and salinity are the two major abiotic stresses disrupting plant growth and productivity [1,2,3]. These stresses are gradually becoming more severe in many places, mainly in arid or semi-arid areas, due to climate change [4,5]. Arid or semi-arid land covers nearly half of the Earth’s land surface and is productive for crop cultivation if irrigation water is available. According to reports, salt affects 20–50% of irrigated cropland [6]. By 2050, approximately half of the world’s arable land will be salinized [7]. The majority of these areas are arid or semi-arid with little precipitation and high evapo-transpiration [8]. Drought is frequently associated with salinity stress in coastal, arid, and semiarid regions. When the soil water evaporates, the salts become concentrated in the soil solution, resulting in combined drought and salinity [9]. Future research should concentrate on the combined stresses because they are crucial for ensuring sustainable agriculture in the era of climate change [10,11].

Salinity causes Na^+^ toxicity and ionic imbalance and disrupts vital metabolic processes in plant cells, such as protein synthesis, enzymatic reactions, and ribosome functions [12,13,14]. High-concentration Na^+^ competes with other essential nutrients such as potassium, magnesium, ammonium, nitrate, and phosphate [15]. However, how drought affects salinity-induced ionic imbalance in plants is not clear. Plant physiological processes are directly or indirectly affected by insufficient water. Photosynthesis is directly inhibited by drought stress [16,17]. Drought reduces morphological and physiological traits, photosynthesis, leaf water potential, sap movement, and stomatal conductance [18]. Moreover, osmotic stress, caused by salinity, impairs the photosynthesis machinery, reducing stomatal conductance, which, in turn, reduces CO_2_ entry, and, ultimately, the rate of photosynthesis [19]. Furthermore, Na^+^ toxicity, caused by salinity, has a detrimental effect on photosynthesis [20]. However, it is unclear which stress, salinity or drought, has a more significant negative impact on photosynthesis. The combined effect of salinity and drought on plant photosynthesis cannot be predicted based on plant responses to individual stresses [21]. Furthermore, the combination of salinity and drought shows additive adverse effect on photosynthesis [22], but the magnitude of the reduction is unknown. In plants, reactive oxygen species (ROS) cause protein denaturation, lipid peroxidation, DNA damage, carbohydrate oxidation, pigment breakdown, and enzymatic activity impairment [23]. Drought-induced stomatal closure reduces a plant’s ability to utilize sunlight and salinity-induced Na^+^ toxicity, resulting in excessive ROS formation in green tissues [12,24]. As a result, salinity and drought stress can cause ROS overproduction [25,26,27]. However, it is still unclear to what extent ROS production occurs when salinity and drought are combined, as well as how the enzymatic antioxidant system responds under combined stress compared to individual stresses.

Overall, changes in plant growth patterns occur when salinity or drought stress disrupts various physiological mechanisms. When salts accumulate in root zones, plants experience physiological drought [28], which affects stomatal physiology and reduces photosynthesis and growth [29]. When soil salinity suddenly rises, leaf cells lose cell volume and turgor [30]. Leaf appearance slows over time, and leaves become smaller [31]. Similarly, drought stress disrupts the plant’s nutrient homeostasis and photosynthesis [32,33]. Plant cells lose turgor under drought stress due to a lack of water, which hampers plant growth [34]. We hypothesized that combined salinity and drought stress adversely affected plants’ physiological mechanisms and growth patterns more prominently than individual stress. However, the quantitative assessment of growth reduction in response to combined drought and salinity compared to individual stress is not well-reported. In this study, we attempted to determine how differentially combined salinity and drought stress and individual stresses affect plant growth. The objective of this study was to investigate a plant’s response to combined drought and salinity stress using a systematic approach. This study compiled findings from 30 different original research publications on the effects of drought, salinity, and their combinations on growth, photosynthesis, oxidative stress, and ionic toxicity (Supplemental file: Table S1).

## 2. Materials and Methods

**Figure 1:**
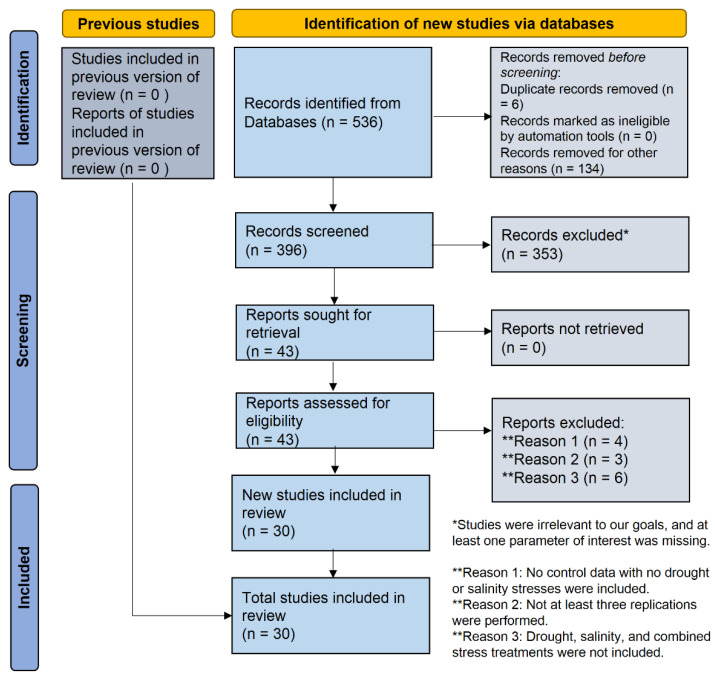
The systematic literature search flow diagram following Preferred Reporting Items for Systematic Reviews and Meta-Analysis (PRISMA). To synthesize and present findings in the current systematic review, we adhered to the PRISMA standards [37].

A total of 30 original research articles were collected in December 2020 via various keyword searches in scientific databases such as Google Scholar, Scopus, and Web of Science. We used the keywords ‘salinity’, ‘salt’, ‘saline’, and ‘NaCl’ for finding salinity-related papers and ‘drought’, ‘water deficit’, and ‘osmotic’ for drought-related papers. We chose research articles with at least one keyword from both drought and salinity in the title. The research papers that matched the selected criteria were identified, and 30 articles out of 43 met the selection criteria. The selection criteria were: (i) the study includes control data with no drought or salinity stresses; (ii) the study includes drought, salinity, and combined stress treatments; (iii) at least three replications were performed; and (iv) at least one parameter of interest was present. Experiments conducted for a short period (<7 days) under stress conditions were omitted. The PRISMA (Preferred Reporting Items for Systematic Reviews and Meta-Analyses) reporting criteria were followed when obtaining the metadata [35,36]. The PRISMA showed the steps of the screening procedures of published articles retrieved from databases (Figure 1). The protocol of this systematic review was registered in the OSF registries (https://osf.io/39s7t).

During the data collection, different plant growth and physiological parameters were considered. The concerned parameters were: plant height (PH), shoot dry weight (SDW), root dry weight (RDW), relative growth rate (RGR), stomatal conductance (G_s_), transpiration (E), net CO_2_ assimilation (A), Na^+^, Cl^−^ and K^+^ contents in the leaves, chlorophyll (Chl) contents (Chl *a, b*, and total Chl) in leaves, superoxide dismutase (SOD), peroxidase (POX), catalase (CAT) and ascorbate peroxidase (APX) activities, malondialdehyde (MDA), and hydrogen peroxide (H_2_O_2_) contents in leaves.

Data for four treatments—control, drought, salinity, and combined salinity-drought—were collected from the selected paper. Figures obtained from papers were digitized using the WebPlotDigitizer 4.2 program (http://arohatgi.info/WebPlotDigitizer/, accessed on 13 June 2021). Some papers were published on more than one genotype; these genotypes were treated as separate case studies.

The method recommended by Cohen et al. [38] was used to conduct the statistical analysis, but we modified our analytical test. Since Welch’s *t*-test performs better when sample size and variances are unequal, we used it instead of the Tukey test. Statistical analyses were performed as the average of all relevant cases because species varied greatly under different stresses (drought, salinity, and combined drought and salinity), and comparing parameters from various papers is not perfectly logical. The findings from every experiment were analyzed as a percentage of the control treatment for all variables. The statistically significant differences were assessed using a two-tailed Welch’s *t*-test. R 4.0.1 was used for all statistical analyses and the visualization of data.

## 3. Results

### 3.1. Effects of Combined Drought and Salinity Stress on Plant Growth

**Figure 2:**
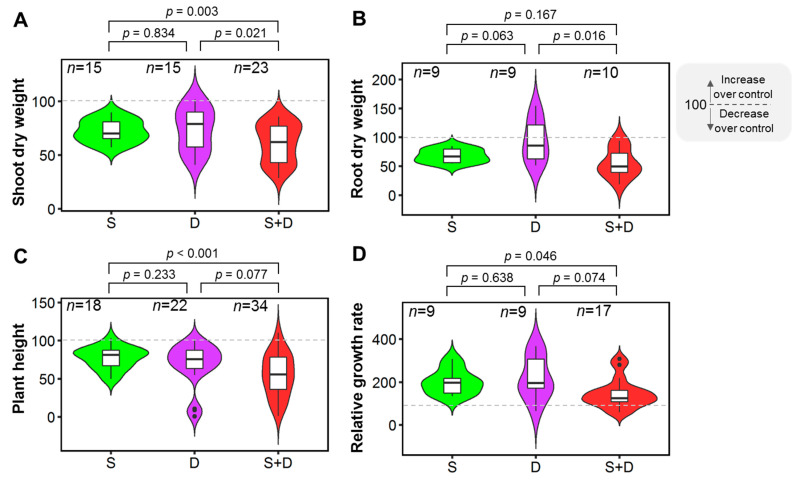
Effects of salinity (S), drought (D), and combined salinity and drought (S+D) stress on growth parameters. (**A**) Shoot dry weight (SDW), (**B**) root dry weight (RDW), (**C**) plant height (PH), and (**D**) relative growth rate (RGR). The % of control treatment is presented in the figures. The statistical differences were assessed using Welch’s *t*-test where the *p*-value indicates the level of statistical difference, and a *p* value less than 0.05 was considered statistically significant. *n* indicates the number of studies.

SDW, RDW, PH, and RGR were used to assess the effects of salinity-drought stress on plant growth-related parameters. Salinity-drought stress had a negative impact on all of these parameters. SDW decreased by 14% and 16% in S+D—stressed plants compared to only salinity- and drought-stressed plants, respectively, which were statistically significant (*p* = 0.003 and *p* = 0.021, respectively) (Figure 2A). Similarly, RDW decreased by 39% in combined-stressed plants compared to drought-stressed plants, which was a significant (*p* = 0.016) difference (Figure 2B). The effects of salinity and S+D on RDW were statistically comparable (*p* = 0.167) (Figure 2B). PH was reduced by 22% in salinity-drought-treated plants compared to salinity-treated plants, which was highly significant (*p* < 0.001) (Figure 2C). The reduction in PH caused by individual salinity and drought treatments was statistically comparable (*p* = 0.233), as well as of individual drought and S+D treatments (*p* = 0.077) (Figure 2C). RGR was significantly reduced under salinity-drought stress conditions compared to salinity stress conditions (*p* = 0.046) (Figure 2D). The effect of individual salinity and drought treatments was statistically comparable (*p* = 0.638), as well as of individual drought and S+D treatments (*p* = 0.074) (Figure 2D).

### 3.2. Effects of Combined Salinity and Drought on Photosynthetic Efficiency

**Figure 3:**
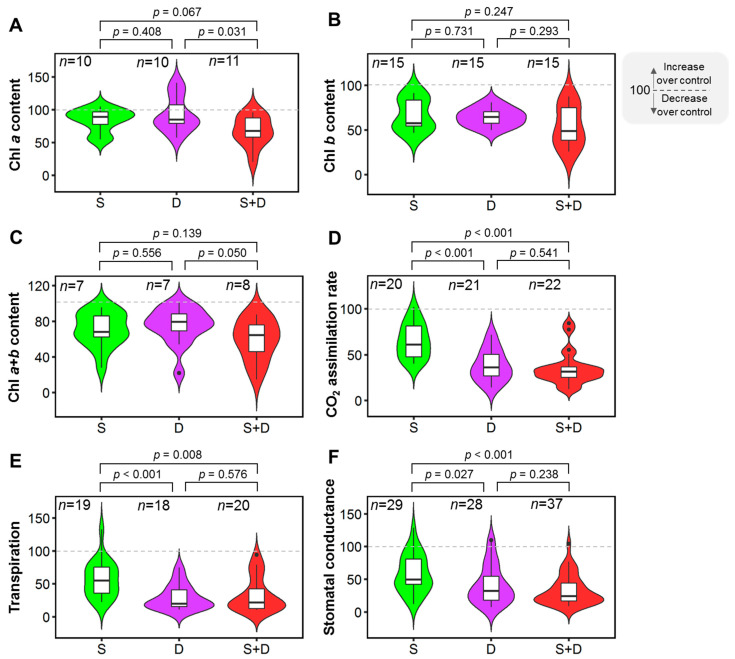
Effects of drought (D), salinity (S), and their combined (S+D) stress on plants’ photosynthetic efficiency. (**A**) Chlorophyll (Chl) *a* content, (**B**) Chl *b* content, (**C**) Chl *a+b* content, (**D**) net CO_2_ assimilation rate (**A**), (**E**) transpiration rate (**E**), and (**F**) stomatal conductance (G_s_). The % of control treatment is presented in the figures. The statistical differences were assessed using Welch’s *t*-test where the *p* value indicates the level of statistical difference, and a *p* value less than 0.05 was considered statistically significant. *n* indicates the number of studies.

Chl *a* content decreased by 25% in salinity-drought-stressed plants compared to drought-stressed plants, a significant (*p* = 0.031) difference (Figure 3A). The reduction induced by individual salinity and drought treatments was statistically comparable (*p* = 0.408), as well as of salinity and S+D treatments (*p* = 0.067) (Figure 3A). The decreases in Chl *b* and Chl *a+b* contents in response to salinity, drought, and salinity-drought stress conditions were statistically non-significant (Figure 3B,C).

A significant (*p <* 0.001) reduction in CO_2_ assimilation rate was observed in S+D-stressed plants compared to only salinity-stressed plants (Figure 3D). S+D and drought had statistically non-significant effects on the CO_2_ assimilation rate (*p* = 0.541) (Figure 3D). Drought-stressed plants had 30% lower A than salinity-stressed plants, a significant (*p <* 0.001) difference (Figure 3D). In response to individual or combined salinity and drought stresses, transpiration rate and stomatal conductance (G_s_) exhibited the same pattern as the CO_2_ assimilation rate (Figure 3E,F). Drought-stressed plants showed a 27% lower transpiration rate than salinity-stressed plants, a statistically significant (*p <* 0.001) variation (Figure 3E). The S+D-stressed plants had a significant (*p* = 0.008) drop in transpiration rate when compared to solely salinity-stressed plants (Figure 3E). The effect of S+D and drought on transpiration rate was statistically insignificant (*p* = 0.576) (Figure 3E). G_s_ decreased by 25% in the S+D treatment compared to the salinity treatment, which was statistically significant (*p* < 0.001) (Figure 3F). The effects of drought and S+D treatments on G_s_ were statistically comparable (*p* = 0.238) (Figure 3F). Individual drought treatments showed 17% lower G_s_ than salinity treatments, a significant (*p =* 0.027) change (Figure 3F).

### 3.3. Effects of Combined Salinity and Drought on Ionic Homeostasis

**Figure 4:**
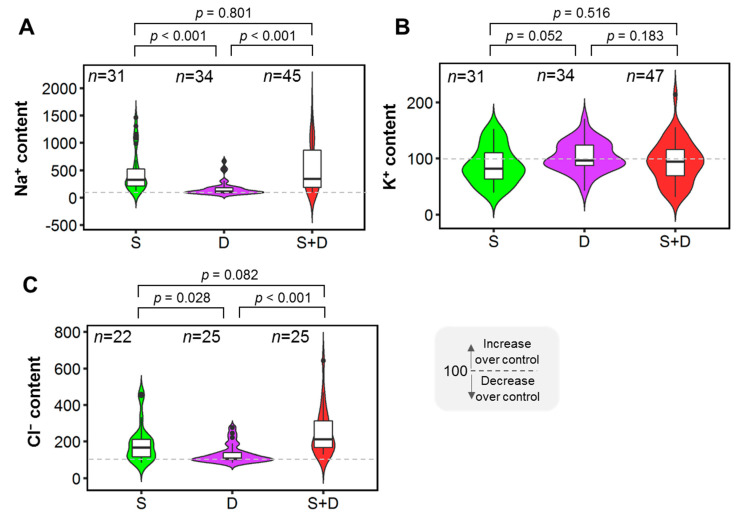
Effects of salinity (S), drought (D), and combined salinity and drought (S+D) stress on ionic homeostasis. (**A**) Na^+^ content, (**B**) K^+^ content, and (**C**) Cl^−^ content. The % of control treatment is presented in the figures. The statistical differences were assessed using Welch’s *t*-test where the *p* value indicates the level of statistical difference, and a *p* value less than 0.05 was considered statistically significant. *n* indicates the number of studies.

Na^+^ and Cl^−^ contents decreased by 347% and 115% in the S+D treatment compared to the drought treatment, with a significant (*p* < 0.001) difference (Figure 4A,C). Furthermore, the Na^+^ and Cl^−^ contents decreased significantly (*p* < 0.001 and *p* = 0.028, respectively) in the drought treatment compared to the salinity treatment (Figure 4A,C). For Na^+^ content (*p* = 0.801) and Cl^−^ content (*p* = 0.082), there were no significant difference between salinity and S+D treatment (Figure 4A,C). The findings revealed no significant changes in K^+^ content under salinity, drought, or S+D conditions (Figure 4B).

### 3.4. Impact of Salinity-Drought on Antioxidant and Oxidative-Related Parameters

**Figure 5:**
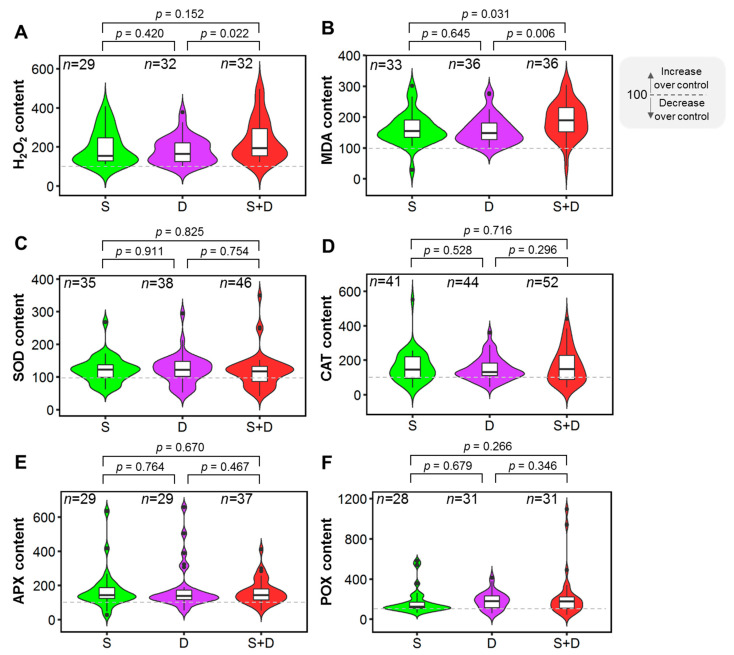
Effects of salinity (S), drought (D), and combined salinity and drought (S+D) stress on antioxidant enzymes and oxidative stress-related parameters. (**A**) H_2_O_2_ content, (**B**) malondialdehyde (MDA) content, (**C**) superoxide dismutase (SOD) activity, (**D**) catalase (CAT) activity, (**E**) ascorbate peroxidase (APX) activity, (**F**) peroxidase (POX) activity. The % of control treatment is presented in the figures. The statistical differences were assessed using Welch’s *t*-test where the *p* value indicates the level of statistical difference, and a *p* value less than 0.05 was considered statistically significant. *n* indicates the number of studies.

Both H_2_O_2_ and MDA levels increased in response to salinity, drought, and S+D stress treatments compared to controls (Figure 5A,B). H_2_O_2_ content increased by 52% in combined S+D-treated plants compared to only drought-treated plants, which was statistically significant (*p* = 0.022). (Figure 5A). On H_2_O_2_ content, the effects of salinity and S+D treatments and salinity and drought treatments were statistically comparable (*p* = 0.152 and *p* = 0.420, respectively) (Figure 5A). MDA content was significantly higher in S+D treatments when compared to salinity (*p* = 0.031) and drought (*p* = 0.006) stress treatments (Figure 5B). The effects of salinity and drought on MDA were statistically comparable (*p* = 0.645) (Figure 5B).

All antioxidant enzymes, including SOD, CAT, APX, and POX, increased in salinity, drought, and S+D stress treatments compared to control (Figure 5C–F). Individual salinity, drought, and S+D stress treatments had no significant effect on SOD, CAT, APX, or POX activity (Figure 5C–F).

## 4. Discussion

Drought and salinity stress reduce crop yield significantly by decreasing plants’ physiological and morphological processes [39,40]. These stresses cause nutritional and ionic imbalances, which have negative impacts on a variety of physiological and biochemical pathways involved in plant growth and development [41]. Researchers are carrying out many studies to investigate the effects of individual salinity and drought, or combined salinity and drought, stress on plants [42,43,44]. The effects of combined salinity and drought stress on several crops, including barley [45], cotton [46], wheat [47], sunflower [21], and maize, have been studied [48]. Overall, these studies have shown that the combined effects of salinity and drought stress have a more significant negative impact on vegetative parameters than their individual effects [49,50,51]. However, no meta-analysis has been performed to determine how much the combination of salinity and drought affects plant growth and physiological and biochemical aspects more than individual stresses. This meta-analysis of 30 papers revealed some new insights into how salinity, drought, and combined salinity-drought stress affect plant stress tolerance in different ways.

Plants generally decline in biomass production when stressed by drought or salinity [52,53]. Multiple research studies found that salinity and drought stress had an additive effect on dry-matter accumulation; thus, the two stresses coupled had a more considerable negative impact [54,55,56]. According to certain studies, salinity causes an increase in the concentration of NaCl, which results in a decrease in shoot length [57,58,59]. However, differing levels of water stress did not significantly impact shoot length, SDW, and RDW [42]. Simultaneous drought and salinity dramatically lowered SDW when compared to salinity or drought alone (Figure 2A). Due to the detrimental effects of drought on both photosynthetic rate and biomass accumulation over the growth period, both the total biomass of plants and the quantity of assimilates declined [38]. Furthermore, the analysis revealed that salinity had a more significant impact on RDW reduction than drought (Figure 2B). When plants are subjected to drought stress, their root length increases mainly as a result of the natural uptake of water and nutrients from deep soil [60]. Since RDW is directly related to root length, drought stress had less effect on RDW reduction than combined stress. When plants are subjected to environmental stresses, they dedicate more biomass production to their roots and enlarge their root system [61]. Combined stress reduced plant height and RGR more than individual drought or salt stress (Figure 2C,D). This could be attributed to a lack of photosynthesis [62,63], as the plant did not acquire enough available water from the soil in the combined stress condition, as the presence of salts under drought conditions enhanced osmotic pressure and also induced ionic toxicity. Such imbalances deleteriously impact various physiological and biochemical pathways involved in plant growth and development [46]. Other proposed explanations for the growth slowdown include reduction in carbon gains and a shift in energy from growth to salt-stress management [64,65].

Research has shown that salt stress significantly reduces chlorophyll concentration [66,67,68]. On the other hand, regular irrigation is connected with the highest chlorophyll content [69]. In general, physiological performance, in particular photosynthetic rate (Pn) and stomatal conductance, increased due to an increase in chlorophyll content because those substances aid in better light absorption. Additionally, a higher amount of light due to chlorophyll increases the probability of Pn because light energy can be converted into chemical energy [70]. Typically, drought stress causes the plant’s chlorophyll content to decrease. Drought stress reduces the chlorophyll content in leaves at various stages of development [71]. Our current study found that combined salinity and drought stress considerably reduced chlorophyll *a* and chlorophyll *a+b* content, while chlorophyll *b* content reduction among treatments was statistically comparable (Figure 3A–C). Furthermore, the fall in chlorophyll content in response to salinity stress was more pronounced than that in drought stress but statistically insignificant (Figure 3A–C). Our results demonstrated that salinity had a substantially more prominent influence on chlorophyll decrease in the presence of drought. This decrease could be attributed to chlorophyll photo-oxidation, their reaction with singlet oxygen, the breakdown of the chloroplast structure, the inhibition of chlorophyll biosynthesis, the destruction of chlorophyll synthesis precursors, the inhibition of new chlorophyll biosynthesis, and the activation of chlorophyll-degrading enzymes such as chlorophyllase [18,68]. The overaccumulation of Na^+^ and Cl^−^ ions caused by salinity has a deleterious influence on chlorophyll concentrations [68].

Stomatal conductance, transpiration, and CO_2_ assimilation rate are all closely related [72,73]. Many researchers have found that salinity [41,74] or drought [75] treatments significantly reduced stomatal conductance and transpiration rate. The accumulation of ions in soil solution increases osmotic pressure, preventing water from being absorbed and transported [76,77], and drought consequently triggers decreased water acquisition in plants [78]. This inhibition causes a cascade of hormone-induced interactions, limiting the rate of photosynthetic activity, CO_2_ assimilation, and stomatal opening [79,80,81]. However, the current study found that combined salinity and drought treatments significantly reduced stomatal conductance, transpiration, and CO_2_ absorption more than the salinity treatment (Figure 3D–F). Single drought stress inhibited photosynthesis more than single salinity [82]. Overall, the results showed that drought stress negatively influences plant photosynthetic properties, which could be related to a lack of water in plants, causing hormonal imbalance. These findings support previous results that net CO_2_ assimilation has little effect under salt stress [83], but combined stress, particularly drought stress, has a significant influence [84].

Na^+^ and Cl^−^ contents were much higher in salinity and combined salinity-drought stress than in individual drought stress (Figure 4A,C), indicating that water constraint in saline soils does not increase Na^+^ and Cl^−^ accumulation in plants. Salinity stress has primarily increased the concentration of Na^+^ while decreasing the concentration of K^+^ [85,86,87], causing the Na^+^/K^+^ ratio in plant cells to fall out of balance [88,89]. Under sustained combined stress, plants encounter ionic toxicity [90,91]. Due to the high concentration of Na^+^, the photosynthetic rate is reduced by stomatal and non-stomatal constraints, notably in the leaf [10]. As a result of the salt stress, leaf and shoot dry weight decreased [92]. Numerous studies have shown that when plants are cultivated in salty soils with or without drought, they accumulate a high concentration of Cl^−^ in their shoot tissues [93,94,95], which concurs with our findings (Figure 4C). Plant growth is inhibited by high concentrations of both Na^+^ and Cl^−^ [96,97], but plants are more sensitive to Cl^−^ than Na^+^ [98]. High Cl^−^ concentrations reduce photosynthetic capacity and quantum yield due to chlorophyll degradation. It could be due to the high Cl^−^ concentration on PSII [98].

Our findings revealed that when plants were subjected to individual salinity or drought stress, as well as combined stress, their H_2_O_2_ and MDA contents increased compared to control conditions (Figure 5A,B). However, MDA and H_2_O_2_ contents increased significantly more under combined stress conditions than under individual stress conditions (Figure 5A,B), indicating that plants experienced an excessive level of oxidative stress under combined salinity-drought stress conditions. Under environmental stress such as drought and salinity, the plant produces excessive ROS; it also produces antioxidants, flavonoids, and secondary metabolites for detoxifying ROS and ensuring protein and amino-acid stabilization under stress conditions [99,100]. Oxidative stress causes oxidative damage by lowering photosynthetic pigments and gas exchange parameters, producing and accumulating ROS [42,101]. Plants contain antioxidant enzymes that protect them from the harmful effects of oxidative stress caused by abiotic and biotic stresses [42,102,103]. Higher antioxidant enzyme activity provides salt and drought resistance by scavenging ROS, and tolerant plants have higher enzyme activities [104,105,106,107]. Under stress conditions, the activities of antioxidant enzymes such as SOD, CAT, APX, and POX were higher than control (Figure 5C–F). Surprisingly, in this study, we discovered that SOD, CAT, APX, and POX activities remained constant in salinity, drought, and their combined stress treatments (Figure 5C–F). This suggests that under severe stress conditions such as combined salinity-drought, the antioxidant enzyme system reaches a steady-state point or becomes exhausted, which is consistent with previous findings [108]. Under severe combined salinity and drought stress, several enzymes (CAT, POX, SOD, glutathione reductase) showed reduced activity [109,110,111]. Concurrently, oxidative damage to the plant is caused by an increase in MDA and H_2_O_2_ [108,111,112]. Many plants showed a greater decline in SOD with an increase in combined stress [102]. Similarly, combining drought and salinity on halophytes (*Halogeton glomeratus*) resulted in significant oxidative damage [108]. Overall, this analysis shows that under combined stress conditions, the exhaustive or steady-state antioxidant system is unable to detoxify the additional amounts of H_2_O_2_, resulting in oxidative damage and eventually reduced growth under combined salinity and drought stress conditions.

During the data collection and from the analyzed results, we pointed out several research gaps. To fill in these gaps and promote future research, we provided several recommendations. The research gaps and recommendations are mentioned below:

- Researchers must conduct more comprehensive genetic and physiological studies to better understand the complex interactions of salinity and drought on plants, including the effects on photosynthesis, plant development, ion concentration, and antioxidant and oxidative-related variables.
- Non-enzymatic antioxidants, including glutathione, ascorbic acid, tocopherols, carotenoids, and others, as well as enzymatic antioxidants, play essential roles in protecting plants from oxidative damage under stress conditions. However, just a few enzymatic antioxidants and nearly no non-enzymatic antioxidants were assessed under combined salinity-drought stress conditions in the selected 30 research articles. As a result, more research is needed to uncover the contribution of non-enzymatic and enzymatic antioxidants in plants’ combined salinity and drought stress tolerance.
- In the present analysis, we found no significant change in K^+^ accumulation and Chl *b* content in leaves between individual and combined stress conditions. More research should be performed to justify these findings and to reveal the putative mechanisms behind that response.
- Sub-group analysis of a dataset could reveal many new insights. For example, how the plant clades, life forms, duration of the life cycle, C_3_ or C_4_, tolerant or susceptible, levels of salinity or drought, plant growth conditions, etc., affect plant responses to salinity and drought stress could be addressed using sub-group analysis. Thus, to address these issues, more studies need to be performed.
- Osmolytes play a crucial role in cellular and plant osmoregulation under individual salinity and drought stress conditions. However, their roles under combined salinity and drought stress have not been reported. Thus, we were unable to include these in this meta-analysis.
- Changes in secondary metabolites in response to combined salinity and drought stress have not been extensively studied.
- Transcriptomics and proteomics analyses should be performed in crop plants grown under individual and combined stress conditions to reveal further insights into combined salinity and drought stress tolerance mechanisms.

## 5. Conclusions

Overall, our findings indicated that combined salinity-drought stress has a greater negative impact on plant growth, photosynthesis, ionic balance, and oxidative balance than the individual stresses. In some cases, salinity had a greater impact than drought stress, and vice versa. Single drought stress inhibited photosynthesis more than single salinity, while single salinity inhibited ionic imbalance more than individual salinity or drought stress. Salinity and drought resulted in an equivalent decrease in shoot biomass, but salinity resulted in a greater decrease in root biomass. The levels of antioxidant systems did not increase in response to combined salinity-drought stress compared to individual salinity or drought stress. Thus, plants experienced more oxidative stress under combined stress conditions. A thorough understanding of plants’ comparative responses to combined salinity and drought stress can help breeders, and plant scientists, develop genetically improved combined stress-tolerant crops. However, the findings of this study could be useful in this regard because it showed how differentially plants respond to combined salinity and drought stress than to the individual stresses.

## Data Availability

The data of the current investigation are available from the corresponding author upon reasonable request.

## Supplementary Materials

The following supporting information can be downloaded at: https://www.mdpi.com/article/10.3390/plants11212884/s1, Table S1: List of papers that were included in the analysis.
