# Supplementary material for: How Do Plants Respond to Combined Drought and Salinity Stress?—A Systematic Review
Source: Plants (Basel). 2022 Oct 28;11(21):2884. doi: 10.3390/plants11212884 (PMC9655390; doi:10.3390/plants11212884)
Supplement: Supplementary file 1 [file plants-11-02884-s001.zip › plants-1920321-supplementary.pdf]

Supplemental File

Supplementary Table S1: List of papers that were included in the analysis.

1.	Goharrizi KJ, Baghizadeh A, Kalantar M, Fatehi F. Combined effects of salinity and drought on physiological and biochemical characteristics of pistachio rootstocks. Scientia Horticulturae. 2020 Feb 5;261:108970.
2.	Siddiqui ZS, Khan MA, Kim BG, Huang JS, Kwon TR. Physiological responses of Brassica napus genotypes to combined drought and salt stress. Plant stress. 2008;2(1):78-83.
3.	Chen, L., Zhang, S., Zhao, H., Korpelainen, H., & Li, C. (2010). Sex-related adaptive responses to interaction of drought and salinity in Populus yunnanensis. *Plant, Cell & Environment*, *33*(10), 1767-1778.
4.	Yang A, Akhtar SS, Li L, Fu Q, Li Q, Naeem MA, He X, Zhang Z, Jacobsen SE. Biochar Mitigates Combined Effects of Drought and Salinity Stress in Quinoa. Agronomy. 2020 Jun;10(6):912.
5.	Hu H, Zhang Z. Individual or Combined effects of Drought and Salinity on Growth and Physiology of Zoysia matrella.
6.	Sun C, Gao X, Fu J, Zhou J, Wu X. Metabolic response of maize (Zea mays L.) plants to combined drought and salt stress. Plant and Soil. 2015 Mar 1;388(1-2):99-117.
7.	Zhen M, Cui M, Xia J, Ma C, Liu C. Effect of nitrogen and phosphorus on alleviation of boron toxicity in Puccinellia tenuiflora under the combined stresses of salt and drought. Journal of Plant Nutrition. 2019 Aug 27;42(14):1594-604.
8.	Umar M, Uddin Z, Siddiqui ZS. Responses of photosynthetic apparatus in sunflower cultivars to combined drought and salt stress. Photosynthetica. 2019 Jan 1;57(2):627-39.
9.	Zhang L, Chen B, Zhang G, Li J, Wang Y, Meng Y, Zhou Z. Effect of soil salinity, soil drought, and their combined action on the biochemical characteristics of cotton roots. Acta physiologiae plantarum. 2013 Nov 1;35(11):3167-79.
10.	Liu Y, Ji D, Turgeon R, Chen J, Lin T, Huang J, Luo J, Zhu Y, Zhang C, Lv Z. Physiological and Proteomic Responses of Mulberry Trees (Morus alba. L.) to Combined Salt and Drought Stress. International journal of molecular sciences. 2019 Jan;20(10):2486.\
11.	Stavridou E, Webster RJ, Robson PR. Novel Miscanthus genotypes selected for different drought tolerance phenotypes show enhanced tolerance across combinations of salinity and drought treatments. Annals of Botany. 2019 Sep 13;124(4):653-74.
12.	Sheteiwy MS, Shao H, Qi W, Hamoud YA, Shaghaleh H, Khan NU, Yang R, Tang B. GABA-alleviated oxidative injury induced by salinity, osmotic stress and their combination by regulating cellular and molecular signals in rice. International journal of molecular sciences. 2019 Jan;20(22):5709.
13.	Ahmed IM, Cao F, Zhang M, Chen X, Zhang G, Wu F. Difference in yield and physiological features in response to drought and salinity combined stress during anthesis in Tibetan wild and cultivated barleys. PloS one. 2013 Oct 24;8(10):e77869.

14.	Ibrahim W, Zhu YM, Chen Y, Qiu CW, Zhu S, Wu F. Genotypic differences in leaf secondary metabolism, plant hormones and yield under alone and combined stress of drought and salinity in cotton genotypes. Physiologia plantarum. 2019 Feb;165(2):343-55.
15.	Sharifian Bahraman A, Sepehry A, Barani H. Plant Responses to Individual and Combined Effects of Abiotic Stresses: Lycium depressum L. Vegetative Parameters under Salinity and Drought. Journal of Rangeland Science. 2020 Jul 1;10(3):228-43.
16.	Dugasa MT, Cao F, Ibrahim W, Wu F. Differences in physiological and biochemical characteristics in response to single and combined drought and salinity stresses between wheat genotypes differing in salt tolerance. Physiologia plantarum. 2019 Feb;165(2):134-43.
17.	Ibrahim W, Qiu CW, Zhang C, Cao F, Shuijin Z, Wu F. Comparative physiological analysis in the tolerance to salinity and drought individual and combination in two cotton genotypes with contrasting salt tolerance. Physiologia plantarum. 2019 Feb;165(2):155-68.
18.	Ellouzi H, Sghayar S, Abdelly C. H2O2 seed priming improves tolerance to salinity; drought and their combined effect more than mannitol in Cakile maritima when compared to Eutrema salsugineum. Journal of plant physiology. 2017 Mar 1;210:38-50.
19.	Ahmed IM, Dai H, Zheng W, Cao F, Zhang G, Sun D, Wu F. Genotypic differences in physiological characteristics in the tolerance to drought and salinity combined stress between Tibetan wild and cultivated barley. Plant Physiology and Biochemistry. 2013 Feb 1;63:49-60.
20.	Ahmed IM, Nadira UA, Qiu CW, Cao F, Chen ZH, Vincze E, Wu F. The Barley S-Adenosylmethionine Synthetase 3 Gene HvSAMS3 Positively Regulates the Tolerance to Combined Drought and Salinity Stress in Tibetan Wild Barley. Cells. 2020 Jun;9(6):1530.
21.	Jamshidi Goharrizi, K., Amirmahani, F., & Salehi, F. (2020). Assessment of changes in physiological and biochemical traits in four pistachio rootstocks under drought, salinity and drought+ salinity stresses. *Physiologia plantarum*, *168*(4), 973-989.
22.	Álvarez, S., & Sánchez-Blanco, M. J. (2015). Comparison of individual and combined effects of salinity and deficit irrigation on physiological, nutritional and ornamental aspects of tolerance in Callistemon laevis plants. *Journal of Plant Physiology*, *185*, 65-74.
23.	Sattar, A., Cheema, M. A., Sher, A., Abbas, T., Irfan, M., Ijaz, M., ... & Ali, Q. (2018). Foliage applied silicon alleviates the combined effects of salinity and drought stress on wheat seedlings. *Int J Agric Biol*, *20*, 2537-2543.
24.	Hussain, T., Koyro, H. W., Zhang, W., Liu, X., Gul, B., & Liu, X. (2020). Low salinity improves photosynthetic performance in Panicum antidotale under drought stress. *Frontiers in plant science*, *11*, 481.
25.	Slama, I., Ghnaya, T., Savouré, A., & Abdelly, C. (2008). Combined effects of long-term salinity and soil drying on growth, water relations, nutrient status and proline accumulation of Sesuvium portulacastrum. *Comptes rendus biologies*, *331*(6), 442-451.
26.	Torun, H. (2019). Time-course analysis of salicylic acid effects on ROS regulation and antioxidant defense in roots of hulled and hulless barley under combined stress of drought, heat and salinity. *Physiologia plantarum*, *165*(2), 169-182.

27.	Umar, M., & Siddiqui, Z. S. (2018). Physiological performance of sunflower genotypes under combined salt and drought stress environment. *Acta Botanica Croatica*, *77*(1), 36-44.
28.	Manuchehri, R., & Salehi, H. (2014). Physiological and biochemical changes of common bermudagrass (Cynodon dactylon [L.] Pers.) under combined salinity and deficit irrigation stresses. *South African Journal of Botany*, *92*, 83-88.
29.	Jin, J., Niu, J., Guo, T., Zhou, R., & Sun, L. Z. (2020). The effect of drought on physiological responses of forage plants to salt stresses depends on occurring time. *Acta physiologiae plantarum*, *42*(6), 1-10.
30.	Slama, I., M'Rabet, R., Ksouri, R., Talbi, O., Debez, A., & Abdelly, C. (2015). Water deficit stress applied only or combined with salinity affects physiological parameters and antioxidant capacity in Sesuvium portulacastrum. *Flora-Morphology, Distribution, Functional Ecology of Plants*, *213*, 69-76.
